# Theragnostic Use of Radiolabelled Dota-Peptides in Meningioma: From Clinical Demand to Future Applications

**DOI:** 10.3390/cancers11101412

**Published:** 2019-09-22

**Authors:** Riccardo Laudicella, Domenico Albano, Salvatore Annunziata, Diletta Calabrò, Giovanni Argiroffi, Elisabetta Abenavoli, Flavia Linguanti, Domenico Albano, Antonio Vento, Antonio Bruno, Pierpaolo Alongi, Matteo Bauckneht

**Affiliations:** 1 Department of Biomedical and Dental Sciences and of Morpho-Functional Imaging, Nuclear Medicine Unit, University of Messina, 98125 Messina, Italy; 2 Department of Nuclear Medicine, University of Brescia and Spedali Civili Brescia, 25123 Brescia, Italy; 3 Institute of Nuclear Medicine, Università Cattolica del Sacro Cuore, 00168 Roma, Italy; 4 Nuclear Medicine, DIMES University of Bologna, S. Orsola-Malpighi Hospital, 40138 Bologna, Italy; 5 Department of Health Sciences, University of Milan, 20142 Milan, Italy; 6 Nuclear Medicine Unit, Department of Experimental and Clinical Biomedical Sciences “Mario Serio”, University of Florence, 50134 Florence, Italy; 7 IRCCS Istituto Ortopedico Galeazzi, Unità di Radiologia Diagnostica ed Interventistica, 20161 Milano, Italy; 8 Sezione di Scienze Radiologiche, Dipartimento di Biomedicina, Neuroscienze e Diagnostica Avanzata, Università degli Studi di Palermo, 90127 Palermo, Italy; 9 Department of Experimental, Diagnostic and Specialty Medicine-DIMES, University of Bologna, S. Orsola-Malpighi Hospital, 40138 Bologna, Italy; 10 Unit of Nuclear Medicine, Fondazione Istituto G. Giglio, 90015 Cefalù, Italy; 11 Nuclear Medicine Unit, IRCCS Ospedale Policlinico San Martino, 16132 Genoa, Italy

**Keywords:** meningioma, somatostatin receptor, neuroimaging, positron emission tomography, radionuclide therapy

## Abstract

Meningiomas account for approximately 30% of all new diagnoses of intracranial masses. The 2016 World Health Organization’s (WHO) classification currently represents the clinical standard for meningioma’s grading and prognostic stratification. However, watchful waiting is frequently the chosen treatment option, although this means the absence of a certain histological diagnosis. Consequently, MRI (or less frequently CT) brain imaging currently represents the unique available tool to define diagnosis, grading, and treatment planning in many cases. Nonetheless, these neuroimaging modalities show some limitations, particularly in the evaluation of skull base lesions. The emerging evidence supporting the use of radiolabelled somatostatin receptor analogues (such as dota-peptides) to provide molecular imaging of meningiomas might at least partially overcome these limitations. Moreover, their potential therapeutic usage might enrich the current clinical offering for these patients. Starting from the strengths and weaknesses of structural and functional neuroimaging in meningiomas, in the present article we systematically reviewed the published studies regarding the use of radiolabelled dota-peptides in surgery and radiotherapy planning, in the restaging of treated patients, as well as in peptide-receptor radionuclide therapy of meningioma.

## 1. Introduction

Meningioma is the most common primary brain tumour, accounting for 36% of all intracranial tumours [1]. Its clinical detection is usually related to the occurrence of symptoms when it reaches a large size [2]. However, with the increasing availability of neuroimaging, incidentally discovered meningiomas are becoming more frequent [3]. The preferred clinical management is heavily based on clinical profile and histopathology. In particular, according to the World Health Organization (WHO) histopathological grading, meningiomas can be classified as benign (WHO I), showing slow growth and a low recurrence rate, atypical (WHO II), or malignant (WHO III), showing a more aggressive clinical behaviour [4,5,6]. The most common treatment options are represented by neurosurgery and, in cases of contraindication, various radiotherapy options, including the emerging heavy particle radiotherapy [7]. However, it has been reported that after gross total resection, 12–19% of all tumours, including WHO grade I, recur within the first two decades [8,9]. Similarly, a tumour control rate varying from 85% to 100% at 5 years has been reported after radiation therapy as a first-line treatment in patients not eligible for surgery, regardless of WHO grading [10,11]. This situation is presumably related to the existence of unfavourable biological variables which, in turn, potentially instigate tumour recurrence, regardless of the histopathological grading. A representative example is represented by radiation-induced meningiomas, in which a higher frequency of malignant features at histopathology or multifocality, as well as higher recurrent-rates, have been reported concerning spontaneous meningioma [12,13]. On the other hand, in many cases, clinical observation with serial surveillance neuroimaging remains the preferred choice, particularly in asymptomatic slow-growing meningioma [3]. However, there is no class I or II evidence to support this convention. Moreover, delaying therapeutic intervention implies renouncing the obtaining of a definitive histopathological diagnosis, at least in the early phases of the clinical assessment, making neuroimaging the only source of information able to impact on clinical decision making. 

For the above-mentioned reasons, in the last years, several molecular, genetic, and neuroimaging biomarkers have been developed, completing the histopathological classification and giving new insights into prognosis and treatment options [14]. Among them, the somatostatin receptor (SSTR) type 2 expression on meningioma cellular surface has recently emerged, with the consequent clinical implication related to the potential use of radiolabelled somatostatin receptor analogues (such as dota-peptides) for both diagnostic and therapeutic purposes. However, SSTR-targeted radionuclide imaging and therapy are, to date, still far from being included in the standard of care of meningioma, as the European Association of Neuro-Oncology (EANO) guidelines have included these approaches only in the “future directions” of meningioma’s clinical practice [15]. In particular, the additive value of SSTR functional imaging with morphological neuroimaging technologies and the exact location of SSTR-targeted radionuclide therapy in the therapeutic flow-chart of meningiomas are still not fully defined. Starting from the strengths and weaknesses of morphological and functional neuroimaging in meningiomas, in the present article we systematically reviewed the published evidence regarding the use of radiolabelled dota-peptides in surgery and radiotherapy planning, in the restaging of treated patients, as well as in peptide-receptor radionuclide therapy. 

## 2. Methods

We searched the PubMed, PMC, Scopus, Google Scholar, Embase, Web of Science and Cochrane library databases (up to June 2019), using the following as both text and as MeSH terms: “dota-peptides”, “somatostatin receptor”, “CT”, “MRI”, “Positron emission tomography” (PET), “PET”, “PET/CT”, “PET/MRI”, and “meningioma”. No language restriction was applied to the search, but only articles in English were reviewed. A further literature search focused on the application of radiolabelled dota-peptides for therapeutic purposes was also performed through the same databases using the following terms: “meningioma”, “PRRT”, “^177^Lu”, “^90^Y”, “^111^In”, “somatostatin”, and “SSTR”, singularly and in cross-reference. Due to the large existing literature about CT and MRI in the diagnosis of meningioma, these articles were not systematically reviewed. Therefore, the corresponding section represents a narrative description of the radiological background for PET imaging in this field. Similarly, a narrative description of PET tracers, other than SSTR-targeted, was included. The systematic literature search returned 354 articles for PET imaging and 270 articles for Peptide Receptor Radionuclide Therapy (PRRT). According to PRISMA flow-chart, after duplicate removal, 24 and 27 articles for PET imaging and PRRT have been considered, respectively. These articles were fully read and analysed according to the title and abstract, as previously described [16]: this approach led to the exclusion of 573 articles for reasons reported in Supplementary Figures S1 and S2. Obtained articles were enough to be discussed separately by the clinical indication.

## 3. Diagnostic Challenges for MRI Imaging in Meningioma

MRI is the modality of choice to image meningiomas, due to its striking contrast resolution and multiparametric characteristics. In addition to MRI, CT has a well-established role in specific cases where calcification and bone changes adjacent to the lesion are present. Several MRI features of meningioma can be helpful to reach the diagnosis, although atypical imaging findings and pitfalls related to artifacts may lead to a wrong interpretation of images, with the diagnosis becoming challenging in some settings. Furthermore, it should be considered that several neoplastic and non-neoplastic conditions may resemble meningioma.

Meningiomas typically appear as extra-axial round or lobulated lesions with well-defined margins and broad dural base. These lesions can be observed along any of the outer surfaces of the brain and within the ventricular system, where they arise from the choroid plexus. These lesions are more frequently located in the parasagittal aspect of brain convexity, the convexity of the lateral hemisphere, the sphenoid wing, the middle cranial fossa, and the olfactory sulcus [17]. Meningiomas generally show homogeneous signal intensity, although many variants with different appearances can be encountered. The typical MRI signal consists of iso-hypointensity relative to grey matter on T1-weighted images and variable intensity on T2-weighted images (ranging from iso to hyperintense). However, they can also appear hypointense on T2-weighted images due to the fibro-collagenous matrix or calcific deposits [17]. In MRI, calcifications are best identified on susceptibility-weighted images as areas of low signal intensity. Nevertheless, calcifications can also be detected on T2-weighted and gradient-echo sequences as intralesional hypointense foci.

Meningioma may occasionally present an aggressive pattern with infiltrating growth over the dura (“en plaque” meningioma), which most commonly occurs along the sphenoid ridge or the convexity. After injection of gadolinium-based contrast agent, MRI images demonstrate strong and homogeneous enhancement of the lesion, which occasionally shows areas of central necrosis or calcifications without post-contrast enhancement. The contrast is especially helpful to delineate “en plaque” meningiomas [18], which generally appear as thickened asymmetric sheets of enhancing dura. Among the MRI findings of meningiomas that lead to the suspect of an extra-axial mass, the “dural tail” sign and the “fluid cleft” are the most relevant. The former results from a thickening of the dura mater, resembling a tail and extending from the mass with homogenous enhancement after contrast media injection [19]. This sign can be helpful, but it is not specific for meningiomas, being also observed in different lesions, such as metastatic lesions, glial tumours, and lymphoma [19,20]. In several cases, especially when meningiomas are located at the skull base, it can be challenging to differentiate the normal aspect of the dura from neoplastic tissue, due to the strong contrast enhancement of both [21]. The latter imaging feature derives from a cleft of cerebrospinal fluid or vessels observed between the tumour and the cerebral cortex. Further, meningiomas are often associated with adjacent bone changes, including osteolysis and hyperostosis [19] with the latter being more frequently observed, especially in the “en plaque form”. In this setting, CT can be more helpful to better depict bone changes related to meningiomas [22]. Nevertheless, it can be challenging to clearly define margins and depth of bone infiltration, despite using CT. Pieper et al. reported that MRI is not sufficiently accurate to demonstrate bone involvement [23]. On the other hand, despite the benign nature of the WHO grade I meningiomas, tumour cells can microscopically infiltrate the adjacent structures, such as vessels and cranial nerves [24,25]. Similarly, primary intraosseous meningioma is a rare benign tumour, mostly arising from the skull bones, but also reported in the nasopharynx or neck [26]. This lesion can be confused with metastasis or other malignant bone tumours, also because its MRI features are relatively non-specific. The lesion appears hypo-isointense on T1-weighted images with strong enhancement in post-contrast images [27]. 

Besides bone involvement, MRI has some limitations in clearly defining the margins of meningiomas, particularly in those lesions with complex geometry and those located in the skull base [15]. In fact, the MRI evaluation of meningiomas can be hampered by artifacts, which are especially encountered in the base of the skull and can partly hide the lesion due to image distortion. Local magnetic inhomogeneities, called “susceptibility gradients”, are more frequently observed in the skull base, where air, bones, and brain are closely associated. These susceptibility artifacts, observed at soft tissue and bone (petrous temporal bones) and air (paranasal sinuses) interfaces, are generally flame-shaped and determine regional image distortion with increased signal intensity [28]. Susceptibility artifacts can also be related to metal bodies and other foreign objects, which can be located “on” and “in” patients’ bodies. These objects can mimic or, conversely, hide pathology, variably reducing images quality. The main problems arise when dealing with those foreign bodies that cannot be removed, especially when located precisely in the site of the lesion (e.g. surgical clips or staplers, endoprosthesis). Other artifacts responsible for a dramatic decrease of image quality can be related to orthodontic appliances and ventricular shunt valves [29].

Another potential source of MRI pitfall is reported as "anatomical blind spots": normal brain regions that are challenging sites to be evaluated with MRI, and where pathologic disorders may be barely detected in imaging examinations [30]. These blind spots include cerebral sulci, dural sinuses, orbits, cavernous sinuses, clivus, Meckel’s cave, brainstem, skull base, and parapharyngeal soft tissues. In these anatomic districts, it is possible to observe densely compact anatomy with the juxtaposition of neural, vascular, bone, and soft tissue structures. In this setting, the detection of small meningiomas can be highly difficult, especially when dealing with small lesions without clear mass effects on the surrounding structures. Clearly, infiltrative lesions, such as small en plaque meningiomas, may also become a diagnostic challenge in these sites.

Finally, another challenging aspect is represented by the post-treatment evaluation, with MRI still having some limitations in the differentiation between residual tumour and recurrent tissue after surgery, as well as between residual tumour and vital tumour from scar tissue after radiotherapy. Accordingly, in the first six months after surgical resection of meningioma, MRI has a decreased accuracy in identifying residual tumour tissue [31].

## 4. Diagnostic Challenges for PET Imaging in Meningioma

Functional studies using FDG-PET/CT showed that meningiomas generally have a low FDG metabolic rate [32]. However, the potential application of FDG-PET imaging to describe meningioma aggressivity, leading to a non-invasive “metabolic grading” has been proposed. In particular, Di Chiro et al. [33] previously reported that FDG accumulation is correlated with the degree of malignancy in meningioma, highly reproducing Thallium-201 accumulation pattern, as depicted by SPECT imaging [34]. Furthermore, Cremerius et al. demonstrated high sensitivity (89%) and specificity (88%) of FDG-PET in detecting high-grade meningiomas [35]. Similarly, Kado et al. previously showed that FDG uptake was high in a case of highly aggressive radiation-induced meningioma [36]. On the clinical ground, FDG positivity in meningioma might become a predictive factor of tumour recurrence at the single-patient level [37]. However, other studies have showed opposite results. Iuchi et al. reported that meningioma FDG uptake was not significantly correlated with Ki-67 index or clinical malignancy [38]. Also, Park et al., who showed a correlation between FDG accumulation and tumour proliferative activity, did not find any capability of FDG-PET to predict tumour grade [39]. For these reasons, further studies are needed to verify the potential clinical application of this tool in the clinical setting.

Because of its low physiological uptake by normal brain, amino acid imaging modalities, such as 11C-methionine (MET) and 18F-fluoroethyl-L-tyrosine (FET), are used for the detection of brain tumours enabling a high tumour/non-tumour contrast. However, the clinical role of these PET tracers in patients with meningioma is still not clear and warrants further investigation. Compared to FDG, MET showed higher detection rates of meningioma both in qualitative and semiquantitative images analysis [40,41]. Its diagnostic applications might be extended to tumour volume delineation for radiotherapy planning [42], as well as to the monitoring of treatment efficacy [43,44]. However, few studies are currently available on the topic. Moreover, while some studies demonstrated a fair correlation between MET uptake and meningioma’s proliferative activity (as assessed by the Ki-67 index [38]), this finding was not subsequently confirmed [45]. Similarly, opposite results were obtained about the correlation between MET uptake and high WHO grade [43,45]. For these reasons, the pathophysiological meaning of MET accumulation in meningiomas, together with its clinical value, still needs to be clarified. Similarly, few studies are currently available on the role of FET-PET in meningioma, including in some cases direct comparisons with SSTR-targeted PET imaging, which shows a more favourable target-to-background ratio offered by the latter approach [46]. However, interesting preliminary data have been published about its potential application in tumour grading [47]. Moreover, differently from MET and SSTR ligands, FET does not accumulate in the pituitary gland, making this tracer potentially superior in detecting intrasellar invasion of meningioma [48,49]. 

Few other tracers have been tested in meningioma, including 11C/18F-choline [50], 18F-Fluoride [49,51], and 11C-Acetate [52]. Again, the small available literature highly prevents their current application in the clinical setting. 

## 5. SSTR-PET in the Definition of Tumour Growth and Tumour Extent for Surgery Planning 

A crucial issue in meningioma management is the detection of tumour growth and extent, which are potentially related to the risk of recurrence and disease progression. 

**Figure 1:**
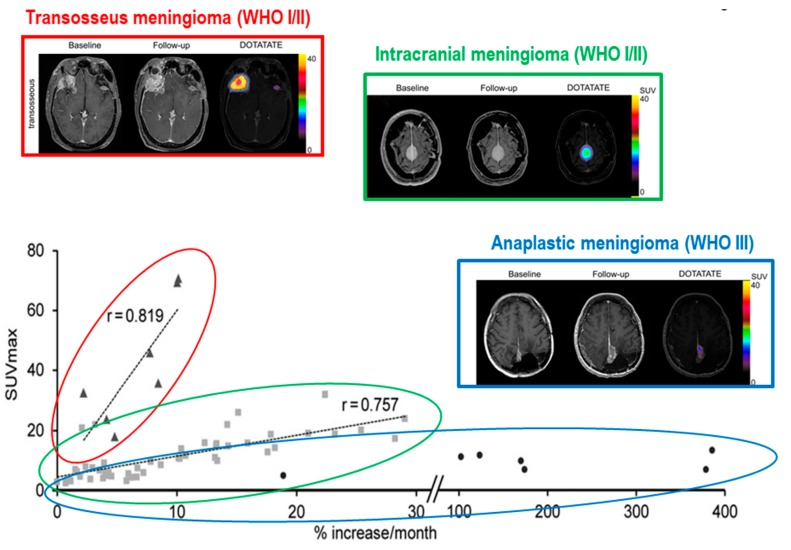
Correlation between SSTR-PET positivity and growth rate in meningioma. The tumour growth rate is directly correlated with SUVmax in grade I and II intracranial meningiomas (green), while this correlation is weak for grade III lesions (blue). As a consequence, high expression of SSTR2 seems to predict faster growth only in well-differentiated meningiomas. Moreover, meningiomas with transosseous growth (red) elicited considerably higher 68Ga-DOTATATE binding. *Adapted with permission from Sommerauer et al.* [59].

The assessment of meningioma’s growth rate is critical to select the appropriate time point for therapy initiation and for the treatment choice. In the clinical practice, the temporal evolution of meningioma, assessed by serial MRI scans, together with the patient’s clinical profile usually guide the surgical approach [15]. Indeed, therapy for patients with meningioma needs to be individualised because of the nature of meningiomas and the potential consequences of different treatments for different patients may vary greatly. However, in some cases, the combination of MRI data and clinic is not enough to define the appropriate treatment strategy. For instance, the growth rates and dynamics among the group of benign meningiomas have been shown to display a huge variability [53,54] and long-term analyses revealed changing growth dynamics in benign meningioma (WHO grade I) with periods of exponential, linear, or no growth, whereas most atypical meningiomas display an exponential growth pattern [55,56,57]. In this scenario, the sole longitudinal observation by successive MRI studies might potentially delay the identification of exponential growth of benign meningioma postponing the timing of surgical resection. As a consequence, several studies have tried to identify novel molecular or imaging biomarkers able to potentially define the tumour growth pattern at baseline, since the time of meningioma identification. Oya et al. [58] identified that the male sex, baseline tumour diameter higher than 25 mm, oedema on MRI, absence of calcifications, presence of symptoms, and T2 signal hyperintensity at MRI, were associated with increased tumour growth in a cohort of 273 intracranial meningiomas. The possible role of SSTR-PET in this field was investigated only in a few papers with promising results but without shared consensus. Sommerauer et al. [59] studied 23 patients with 64 meningioma who underwent 68Ga-DOTATATE PET/CT showing that tracer uptake (measured as the maximum standardized uptake value, SUVmax) is significantly correlated with tumour growth rate in WHO grades I and II meningioma, whereas no association was observed in WHO grade III lesions (perhaps due to the dedifferentiation phenomenon of aggressive meningioma cells that lost the expression of somatostatin receptors, Figure 1). Using multivariate analysis, SUVmax was the strongest predictor of the subsequent tumour growth even including baseline tumour diameter. These results demonstrate that SSTR2 expression predicts tumour growth in well-differentiated meningioma, suggesting the potential role for SSTR-PET in planning the better time point for surgical resection. This could be clinically relevant in meningioma diagnosed in critical sites in which SSTR positivity might guide the surgical resection, even in small, asymptomatic, well-differentiated lesions. 

On the other hand, in the same study, higher SSTR positivity was found in transosseous- vs. intracranial-growing lesions [59]. This finding configures the possible role for SSTR-PET as a further guide to surgical resection, by defining the exact tumour extent. Similarly, Kunz et al. [60] studied the osseous involvement with pathology-proven bone infiltration as the standard of reference, with 68Ga-DOTATATE PET/CT and contrast enhanced MRI both qualitatively and quantitatively. Using PET quantitative analysis, the authors confirmed the adequate distribution of the tracer in intraosseous components of meningiomas, with significantly higher tumour-to-background signals than MRI. Moreover, PET/CT provided a significantly larger volume estimate for the intraosseous extent, while the estimated volumes for extraosseous meningiomas did not differ significantly between the two imaging techniques. But, the high sensitivity of SSTR-PET imaging in the definition of tumour extent is not restricted to the bone invasion. Afshar–Oromieh et al. [61], studied 134 patients with 190 meningioma undergoing both 68Ga-DOTATOC PET/CT and MRI, showing that SSTR-PET provides better sensitivity in the overall detection of meningioma, especially in case of tumours adjacent to the falx cerebri, at the skull base, or in presence of calcifications, which reduce MRI accuracy, as stated above. Rachinger et al. [62] found that the threshold for best discrimination between tumour and tumour-free tissue as an SUVmax equal to 2.3 by spatially precise neuro-navigated tissue-sampling during surgical resection in 21 adult patients with primary or recurrent meningiomas, prospectively enrolled. Using this cut off, tumour tissue was identified with higher sensitivity than MRI (90% vs. 79%), without risking overtreatment, an aspect desirable in an imaging modality used for treatment decision making. Altogether, these data strongly support the use of SSTR-PET imaging for tumour delineation in meningiomas with suspected osseous infiltration, as well as in regions such as the skull base, orbits, or cavernous sinuses, where biopsy has a high haemorrhagic risk, and radiological images are often unclear. However, a limitation related to the use of PET SSTR-ligands may be the study of parasellar region, because the pituitary gland is the intracranial site showing higher physiologic SSTR2 expression. Although Henze et al. [63] showed a divergent dynamic uptake between meningiomas and pituitary gland after injection of 68Ga-DOTATOC, SSTR-PET accuracy might be reduced in this site. 

## 6. SSTR Radio-Guided Surgery

Radio-guided surgery (RGS) offers several theoretical advantages in meningiomas, providing a more accurate detection of tumour lesions and of the total tumour burden “marked” by the radiopharmaceutical. Moreover, since radical resection is crucial for prognosis, RGS might allow confirmation of margins negativity. This is potentially relevant in the case of meningiomas “en plaque” and cranial base meningiomas that often involve cranial base bone [64]. However, up to now, few studies have evaluated the potential clinical benefit of RGS in meningiomas.

Gay et al. [65] showed that SSTR-targeted RGS is feasible and could increase the probability of complete meningioma resection in a cohort of 18 patients with “en plaque” meningiomas who underwent pre- and post-operative SSTRs imaging. Gamma probe intraoperative detection was performed 24 h after the intravenous administration of 111In-DTPA octreotide. In a subgroup of patients, detection of dural or periorbital involvement of “en plaque” sphenoid wing meningiomas was more difficult due to technical reasons, mainly related to the size of the device. Eight patients underwent postoperative scintigraphy, which resulted in a negative, confirming the radicality of the surgical resection. However, in two cases a slight detection three months after the surgical removal was observed, potentially related to post-surgical inflammatory tissue [65].

β- emitting RGS represents a potential further development: β- radiation millimetric tissue penetration, with essentially no γ contamination, better delineates the margins of the lesion, requiring lower activity than traditional γ-RGS and so rendering medical personnel radiation exposure negligible [66]. Collamati et al. used a dedicated β- probe and demonstrated a high target-to-background (TBR) in 10 of 11 patients with meningioma using RGS with β- emitters (TBR > 10 in almost all cases, and usually above 20). These results suggest that the technique can work even with administered activities much smaller than those needed for diagnostic PET scans [67].

Although promising, RGS is not routinely used and further studies are needed to better understand its clinical impact and feasibility of beta-probes employment.

## 7. SSTR-PET in Radiotherapy Planning

In the multimodal approach to meningioma, radiotherapy (RT) is a central treatment alternative to surgery [15]. Since surgery can result intricate due to the complex anatomy of the skull-base region, RT on the surgery bed, residual lesions or not resectable lesions has been associated with good local control and low toxicity. Recently, more and more high-precision RT techniques have been developed, like fractionated stereotactic radiotherapy (FSRT), intensity-modulated radiotherapy (IMRT), stereotactic radiosurgery (SRS), and particle therapy with protons or carbon ions. Current standard imaging for meningioma RT planning is contrast-enhanced MRI co-registered with CT. Post-contrast T1-weighted MRI is usually applied to delineate meningioma lesions and soft tissue, associated with CT scan for better bone assessment. However, the more precise RT techniques have become, the more precise tumour delineation must be. Defining gross tumour volume (GTV) can be challenging in those cases of postoperative settings, equivocal bone thickening, or enhancing dura tails. Thanks to the high tumour-to-background contrast over the past ten years, several studies have reported about integrating SSTR-PET in RT-planning, resulting in additional information on tumour extension, reduction on dose absorbed to organs-at-risk (OAR), and modification in final volume treated.

The first experience was provided by Milker-Zabel and colleagues in a study conducted on a population of 26 patients with WHO grade I to III, most of who had a recurring lesion or received previous radiotherapy treatment. Applying 68 Ga-DOTATOC-PET/CT to the planning target volume (PTV) for FSRT caused significant modifications in 73% of patients, leading to a larger final PTV in 10 out of 26 patients and a smaller one in 9 out of 26 patients [68]. These data were subsequently confirmed by Gehler et al. in a retrospective analysis on 26 patients selected for skull base meningioma IMRT planning. PET imaging provided additional information in 17 patients (65%). Overall GTV based on MRI/CT was larger than GTV-PET in 10 patients and smaller in 13 patients. Since a validated cut-off value for the SUV of DOTATOC was missing, PET window levels were adjusted so that the PET tumour delineation optimally matched with a discernible tumour on MRI/CT [69]. The same approach was adopted by Nyuyki et al. on a definitive population of 39 meningioma patients, where SSTR-PET led to a modification in 28 out of 39 cases (GTV-PET larger than GTV-MRI/CT in 49%, smaller in 23%) [70]. Graf et al. retrospectively evaluated the impact of DOTATOC PET in 48 skull base meningioma patients, integrating PET information in FSRT planning, and showing that PET resulted in major changes in 32 cases (67%). Again, final GTV was smaller than conventional GTV-MRI-CT in 40 meningiomas (83%) and larger in 6 [71].

About particle RT, Combs et al. prospectively planned with MRI and DOTATOC PET, proton and carbon ion therapy of 70 patients with meningioma of heterogeneous pathological grading. The addition of PET provided valuable information in all patients and a reduction in GTV final volume in 40% of cases. These volumes in MRI were extending into soft tissue, like pterygoid muscles, pharyngeal structures. By contrast, PET volume contouring in smaller lesions of the skull base or located within the cavernous sinus or spheno-orbital bony region led to an increase in GTV [72]. Madani et al. investigated the feasibility of dose painting in IMPT based on 68Ga-DOTATATE-PET/CT in 5 patients treated with proton beam-therapy. In this experiment, GTV-PET and GTV-MRI/CT were determined by auto-segmentation with a 50% threshold SUVmax on PET scans and traditional manual contouring on MRI/CT respectively, resulting in a quite small PTV-PET [73].

**Figure 2:**
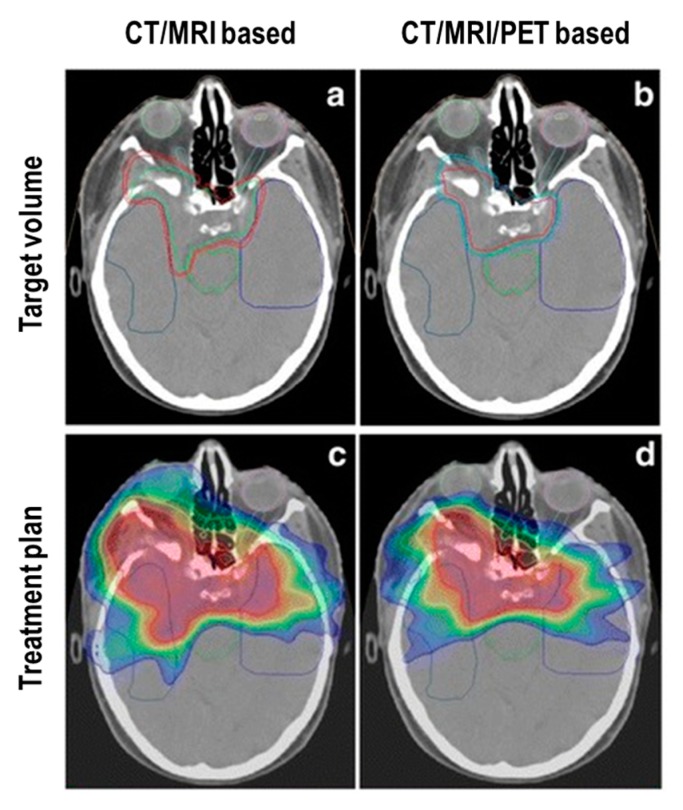
Reduction of IMRT target volume provided by adding 68Ga-DOTATOC-PET to CT and MRI. Panel (**a**) and (**b**) display the target volume based on CT and MRI and CT, MRI as well as 68Ga-DOTATOC-PET, respectively in a patient treated for a skull base meningioma. Panel (**c**) and (**d**) show the corresponding IMRT treatment plans. The present case shows the reduction of dose to OAR by adding SSTR-PET imaging to treatment planning. *Adapted with permission from Stade et al.* [74].

Stade et al. [74] focused their study on the adsorbed dose to OAR. A retrospective analysis was performed on 10 patients randomly selected from an institutional database. Both treatment plan for IMRT and proton therapy were evaluated on an MRI/CT and 68Ga-DOTATOC PET basis. Doses to relevant OAR were calculated (brain stem, optic chiasm, left and right optic nerve). Implementing PET in planning definition achieved a relevant decrease of the extension of target volumes compared to MRI/CT volume definition. More importantly, OAR doses were significantly reduced by PET, especially in the IMRT setting [74] (Figure 2).

Altogether, the above-mentioned experiences suggest that DOTATOC-PET scans have high meningioma-to-background contrast, and that functional imaging provides a better delineation between an area of normal tissue and meningioma residual, bone infiltration, or dural tail leading to additional information or modification on RT-planning, especially for skull base meningiomas. However, a limitation when comparing results of those publications is caused by interobserver variability due to different methods applied on tumour contouring and windowing. Since a validated SUV cut-off value was missing, PET window levels were adjusted so that the PET tumour delineation optimal matched with a discernible tumour on MRI/CT by most of the authors, whereas others decided to apply an arbitrary percentage threshold to SUVmax. Based on these discrepancies, Maclean et al. tested the interobserver variability in a pilot study conducted on 10 patients who underwent simultaneous 68Ga-DOTATATE PET/MRI followed by PET/CT. They were selected as it was anticipated that target volume definition in their cases would be particularly challenging. Three radiotherapists applied an agreed protocol to contour target volumes. Statistical evaluation resulted in significant interobserver variability, particularly in bone followed by dural tail, postoperative bed, and venous sinuses. Nevertheless, absolute volumes contoured between different observers were often similar. Tumour edges evaluated with PET/MRI were sharper than PET/CT ones, but this did not affect contouring. Authors’ advice is that PET-based RT contouring may have a role only in selected cases of meningioma where tumour boundaries are unclear on MRI and CT [75]. Conversely, the preliminary results of the group of Acker et al. on the evaluation of PET/MRI in SRS planning, suggest a significant impact in contouring lesions, particularly when the operator is at the beginning of his learning curve. Authors retrospectively selected 10 patients of which SRS-planning was done by 3 radiosurgeons (RS0, RS1, RS2), one of them (RS2) less experienced than the others. One of the most relevant analyses was the difference between the percentage of the volume defined by RS0 that would not have been treated if RS1 and RS2 had contoured without PET, 19% and 40.2% respectively, suggesting how the extent was highly dependent on operator experience [76]. However, further investigations conducted on larger populations are needed to establish a standardized protocol for windowing PET images over MRI in the definition of target volume contouring in order to harmonise the inter-centre analyses.

## 8. SSTR-PET in Restaging of Treated Patients 

Although meningiomas are usually histologically benign, recurrence and progression are not infrequent. Indeed, distinguishing viable tumour from scar tissue by CT or MRI alone could be challenging, especially in cases of scar formation after surgery or radiation therapy [77], where MRI fails to differentiate between radiation-induced necrosis and recurrent disease in many cases. In fact, the main features of recurrent brain tumours are typically intravenous contrast enhancement, mass effect, and associated vasogenic oedema. On the other hand, post-treatment necrosis has similar characteristics, making it difficult to reliably distinguish from tumour recurrence. In this context, SSTR-PET imaging can play a fundamental role in the visualization of residual meningioma tissue, considering the high levels of SSTR-2 expression in meningiomas and the favourable target-to-background ratio [78]. Indeed, Rachinger and colleagues demonstrated the high sensitivity of 68Ga-DOTATATE in the discrimination between scar tissue and residual tumour due to low uptake in bone and healthy brain tissue. In particular, PET imaging showed a higher sensitivity (90% vs. 79%), with specificity and positive predictive values similar to MR imaging, for both de novo and recurrent tumours. The combination of superior sensitivity with a non-inferior specificity proved that SSTR-PET provides additional useful information that might help to overcome the MRI critical limitations in the discrimination between scar tissue and recurrent tumour tissue [62]. More recently, in a retrospective study of 20 patients, Ivanidze et al. [79] demonstrated superior PET/MRI soft-tissue resolution, during the simultaneous acquisition of PET and MRI imaging, especially for the clinical purposes of differentiating recurrent meningioma from post-treatment changes. They used the superior sagittal sinus as a background reference region, given its absence of SSTR2. They showed a significant difference in the meningioma/reference ratio compared to posttreatment-change/reference ratio. 

**Figure 3:**
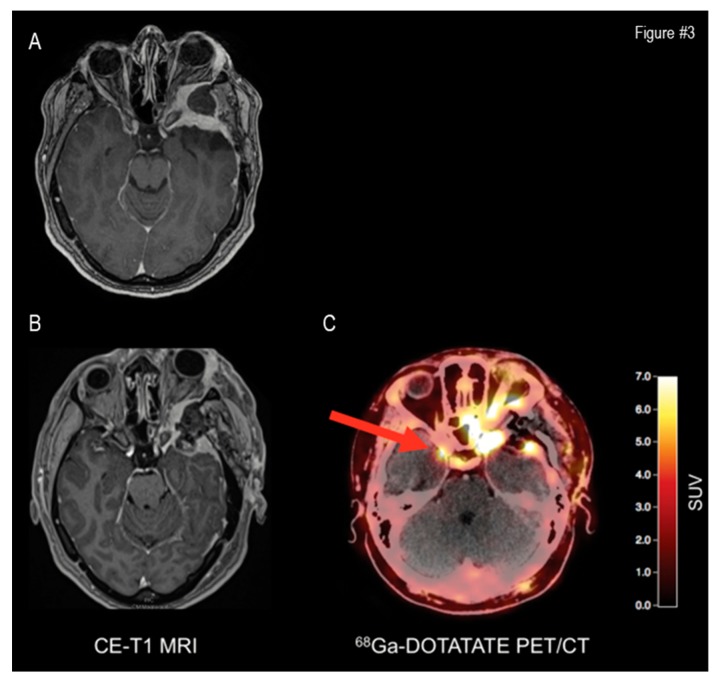
Additive value of SSTR-PET to MRI in restaging. A 42-year-old patient with a history of a left sphenoid skull base meningioma. Preoperative MRI shows tumour recurrence (Panel **A**). Postoperative MRI (Panel **B**) shows an incomplete resection of the tumour, necessitating adjuvant radiotherapy. 68Ga-DOTATATE PET/CT reveals an additional meningioma located at the tip of the right sphenoid wing (Panel **C**). *Reprinted with permission from Galldiks et al.* [78].

Another limitation of morphological imaging is the identification of the transosseous extension of meningiomas, which is of utmost importance in the restaging of treated patients. As previously mentioned, contrast-enhanced MRI has limited sensitivity in the diagnosis of intracranial meningioma bone involvement, where the detection of osseous infiltration relies on few and non-specific morphologic features, such as hyperostosis or intraosseous contrast enhancement. Kunz et al., in a recent study, demonstrated the limited sensitivity of contrast-enhanced MRI to detect bone involvement in intracranial meningiomas in both pre- and post-operative phases, compared to the 68Ga-DOTATATE PET study. They showed the better diagnostic performance of SSTR-PET for treated lesions compared to standard MRI (sensitivity, 97% vs. 54%; specificity, 100% vs. 83%; positive predictive value, 100% vs. 95%; and negative predictive value, 86% vs. 23%) [60]. A representative case of improved restaging by SSTR-PET with respect to MRI is represented in Figure 3.

## 9. Radionuclide Therapy in Meningioma

As stated before, the first therapeutic choice in meningioma patients is represented by the surgical resection. However, for several reasons, complete resection is not always possible. Thus, other therapeutic opportunities in such patients include radiation therapy, SRS, heavy particle therapy, chemotherapy, immunotherapy, somatostatin therapy, and PRRT [80]. The molecular target used for PRRT is usually also labelled with a diagnostic radionuclide, or administered at different doses, to image patients before (demonstrating an adequate lesion’s SSTR expression, that should be greater than the hepatic background uptake), during and after treatment [81]. The aim of creating such a “theragnostic” agent, is to couple the targeted therapy with diagnostic imaging using scintigraphy, SPECT, SPECT/CT, PET/CT and/or PET/MR to quantify the target expression in order to select patients that might most likely benefit from this treatment, also enabling the biodistribution assessment after each cycle of PRRT. 

The origin of PRRT dates back to 1992, when, for the first time, a patient with a primary pancreatic islet cell tumour (glucagonoma) expressing SSTRs was successfully treated with high doses of 111Indium(111In)-penteotride, using the specific physical characteristics of the Auger’s emission and conversion electrons of 111In [82]. However, due to its short tissue’s range resulting in modest tumour shrinkage, it became clear that 111In was not the most suitable radionuclide for PRRT. During the same period, the dota-chelated peptides started becoming available, making it easier to label beta-emitter conjugates [83,84]. Thus, currently, the most used radiometals for therapy are beta-emitters 90-Yttrium (90Y) and 177-Lutetium (177Lu), typically administered in depot formulations every 6 to 10 weeks. Beta-emitters have a short linear energy transfer (LET) and a long range of tissues’ penetration, therefore increasing the average dose delivered to the tumour, but also to the surrounding healthy tissues (90Y more suitable for large-volume disease, 177Lu for small-volume lesions) with a low-incidence of mostly reversible and mild secondary effects, mainly hematological (more frequent with 177Lu) and renal. Namely, in the literature there is an overall risk of 2% for developing myeloproliferative disorders [85]; however, due to the mainly renal excretion of these tracers, renal toxicity is the most limiting factor over time, even if aminoacidic infusion notably reduces the radiopharmaceutical’s renal uptake [86,87], thus narrowing this event to other therapies (i.e., chemotherapy, chemoembolization, radioembolization, and so on). In order to reduce toxicity while preserving the absorbed-effective dose, two principal strategies have been described using a variable activity in four cycles to reach the dose limit [88] or a fixed 7.4 GBq for each cycle until the achievement of the biological effective dose (BED) limit for at-risk organs [89]. 

To date, 177Lu radionuclide therapy is mainly used to treat patients with neuro-endocrine tumours (NET) targeting SSTRs [90], and patients with metastatic castration resistant prostate cancer targeting prostate-specific membrane antigen (PSMA), which is expressed at high levels on the surface of prostate cancer cells (radioligand therapy, RTL) [91]. However, with their localization outside of the blood-brain barrier and high SSTRs expression, meningioma also represents ideal targets for PRRT, that in this setting has been mainly used on a compassionate basis [67,92]. Despite the heterogeneity of the studies in the literature, PRRT in such patients reached a condition of stable disease (SD) in most WHO grade I patients, with a 6-months progression-free-survival (PFS) ranging from 55% to 100%, and variable results in WHO grade II and III. In this paragraph, we focused our attention on the main results of the major studies published for each radiopharmaceutical related to this topic (see also Table 1 for the main characteristics of PRRT radiopharmaceuticals and Table 2 for the main characteristics of the analysed papers).

### 9.1. 90-Yttrium

For many years, 90Y-octreotide was widely used as the radiometal of choice for PRRT [102]. One of the first experiments using 90Y-DOTATOC was done in Basel in 1999 in a cohort of 29 patients (3 of them affected by meningioma): they were treated with four or more doses of 90Y-DOTATOC with ascending activity at intervals of approximately 6 weeks (cumulative dose 6.12 ± 1.3 MBq/m^2^). Treatment was monitored by CT and 111In-DOTATOC scintigraphy. According to 111In-DOTATOC scintigraphic uptake, 20 out of 29 patients showed SD (2 out of 20, meningioma patients), 2 out of 29 showed partial remission (PR) (decrease of ≥50% in tumour volume on CT scans), 4 a reduction in tumour mass <50% (1 of them affected by meningioma), and 3 developed a progressive disease (PD) [103]. More recent studies demonstrated that 90Y-PRRT can slow meningioma growth in the absence of any major adverse events. Bartolomei et al., in a cohort of 29 meningioma patients, despite a non-significative reduction in lesion size, reported (at 3 months after PRRT completion) SD in 19 out of 29 patients (66%) and PD in 10/ out of 29 (34%), with a median PFS of 61 months in WHO grade I vs. 13 months in WHO grade II and III groups. Moreover, the authors reported a median overall survival (OS) of 69 months for WHO grade I vs. 30.5 months for WHO grade II and III patients [93]. In another study on a population of 15 patients with recurrent or progressive meningioma (9 out of 15 WHO grade I) treated with 90Y-PRRT, a condition of SD was obtained in 13 patients (86.7%) and PD in 2 patients (13.3%), with an overall median PFS of at least 24 months (range 0–137) [94]. 

### 9.2. 177-Lutetium

In 2000 another radiometal became available: 177Lu-DOTATATE. Due to a lower tissue penetration range (lower dosimetry to kidneys) and its photon gamma co-emission (that allow having both scintigraphic images and dosimetric studies at the same time), 177Lu became the radiometal of choice over 90Y for PRRT. During the last decades throughout Europe, the number of patients treated with 177Lu-DOTATATE dramatically increased. One of the first experiments was described by the group of Van Essen et al. with 177Lu-DOTATATE therapy in 5 patients with high-grade cranial or cervical meningioma, reporting PD in 3 patients and SD in 2. Moreover, the authors affirmed that if PRRT is used earlier or in combination with other therapies, results might be better [99]. Sabet et al. described the effects of 177Lu-DOTATE PRRT in a patient with pulmonary metastasis from anaplastic meningioma, reaching a dramatic reduction of symptoms with an improvement in the quality of life (SD at 3 months) [100]. Furthermore, Kreissl et al. described their experience in 10 meningioma patients treated with either 177Lu-DOTATATE (4 out of 10) or 177Lu-DOTATOC (6 out of 10) and external beam-RT. The authors described complete response (CR) in 1 patient, SD in 8 patients, and PD in 1 patient. Further, they reported that lesion volume after therapy (as estimated by 68Ga-PET/CT imaging) became 81% ± 21% of the baseline volume [101]. In a recent study about a small cohort of meningioma patients treated with 177Lu-DOTATATE, Parghane et al. reported the occurrence of PR in 3 out of 5 and PD in 2 out of 5 at 68Ga-DOTATE PET/CT follow-up, while, at the structural imaging response evaluation, the authors observed a condition of SD in 100% of patients (5 out of 5), with a mean PFS of 26.25 months (in absence of any major adverse events) [97]. 

### 9.3. Miscellaneous

Minutoli et al. reported their experience in 8 patients treated with PRRT with 111In-Pentetreotide alone and in association with 90Y or 177Lu. In the absence of any major adverse events, the authors observed PR in 2 patients (25%), SD in 5 (62.5%), and PD in 1 (12.5%) [80]. In a long-term study of a cohort of 34 meningioma patients treated with 90Y and 177Lu, Marincek et al. described a condition of SD in the 68% (23/34) with a mean survival of 8.6 years, considering the time of recruitment. Furthermore, SD after treatment and high tumour uptake were significantly associated with longer survival [95]. In the study by Seystahl et al., PRRT (with 177Lu and/or 90Y) led to SD in 50% of population (10 of 20 meningioma patients), with, respectively, a median PFS of 32.2, 7.2, and 2.1 months for G1, G2 and G3, and a median OS of 17.2 months in G3 patients (not reached for G1–2). Furthermore, the authors affirmed that in a 68Ga-PET/CT lesions-based analysis pre-PRRT, SUVmax and SUVmean were significantly higher in those lesions with radiological stability after 6 months and SSTRs expression (at immunohistochemistry) was related with a PFS of > 6 months [96].

## 10. Conclusions and Future Perspectives

Thanks to the high sensitivity of SSTR detection, SSTR-targeted molecular imaging might improve several clinical challenges in meningioma from the definition of tumour growth rate and tumour extent, to the guide of therapy and, finally, to the restaging of treated patients, extending beyond the limitations of morphological imaging in these settings. Similarly, SSTR molecular imaging represents the fundamental prerequisite for SSTR-directed PRRT, which has emerged as a well-tolerated treatment option in patients with advanced recurrent or refractory meningioma, stabilizing or decelerating tumour growth. 

However, several issues should be solved in the near future to further validate the additive value of these approaches in the clinical setting. First, in many cases, analysed studies were conducted on small populations. Larger prospective studies are needed to further verify the capability of SSTR-targeted imaging to significantly impact clinical decision making with respect to conventional imaging. Second, the integration between SSTR-imaging with MRI is still to be explored in the clinical setting. In particular, it could be foreseen that the introduction of the hybrid SSTR-PET/MRI approach will represent the ideal combination of radiological and nuclear medicine strengths, leading to high sensitivity, specificity, and diagnostic accuracy for meningioma, but further studies are needed to confirm or controvert it. In a pivotal study by Afshar-Oromieh et al. [104] the feasibility of 68Ga-DOTATOC PET/MRI for the detection of intracranial meningiomas was analysed in a cohort of 15 meningioma patients. The authors demonstrated that 68Ga-DOTATOC PET/MRI could represent the ideal combination of sensitivity and specificity, offering the best morphological visualization together with SSTR expression status. However, the authors also showed the absence of superiority in treatment planning with respect to the current diagnostic gold standard. Similarly, if the global reduction in the final radiation exposure represents an acknowledged advantage provided by PET/MRI (MR-based attenuation correction allows to eliminate the patient’s radiation exposure deriving from CT [79]), financial benefits, both for the patient and the health service, still need to be demonstrated. 

## Figures and Tables

**Table 1:** Characteristics of PRRT Main Radiopharmaceuticals.

Radionuclide	Physical Half-Life	Emission Peaks Energy	Main Applications	Decay (Abundance)	Penetration Range	Source of Production	Specific Activity
111-Indium (^111^In)	67.9 h	γ (173 keV) γ (247 keV)	Imaging	Electron Capture (100%)	0.002–0.5 mm	Cyclotron	Medium
90-Yttrium (^90^Y)	64 h	β^−^ (2288 keV)	Therapy	β^−^ (100%)	4–8 mm	Generator or reactor	High
177-Lutetium (^177^Lu)	6.7 days	β^−^ (500 keV) γ (208 keV) γ (113 keV)	Only in therapy	β^−^ (100%) γ (27%)	1–2 mm	Reactor	Medium/high

**Table 2:** Results of the Main Papers about PRRT in Meningioma.

Author [Ref]	Meningioma (Total Cohort)	Year	Therapy	Cycles	Total Activity (Gbq)	Type of Response	PFS after PRRT (Months)	Previous Treatment	FU (Months)	Other Main Results (If Present)
Bartolomei et al. [93]	29 (29)	2009	90Y-DOTATOC	2–6 (range)	5–15 (range)	SD 19 PD 10	61 (median for G1) 13 (median for G2-G3)	Surgery 26 RT 18 CT 1 CT + RT 1	3 (for response assessment) 4–77 (range)	Median OS was 40 months. Stabilization of neurological symptoms in 41% until 1 year from last PRRT.
Gester–Gillierson et al. [94]	15 (15)	2015	90Y-DOTATOC	2–4 (range)	13 (median) 1.35–14.8 (range)	SD 13 PD 2	24 (median) 0–137 (range)	Surgery 6 NA or naive 5 Surgery + RT 3 Surgery + RT + CT 1	49.7 (mean) 12–137 (range)	Hematologic, neurologic, and renal toxicities were transient and moderate.
Marincek et al. [95]	34 (34)	2015	90Y-DOTATOC 177Lu-DOTATOC	1–4 (range)	1.5–18.3 (range for Y) 7.4–22.2 (range for Lu)	SD 23 PD 11	NA	Surgery 25 CT 11 RT 1	21.8 (median) 1–137.4 (range)	PRRT may improve the quality and longevity of life with no significant complication.
Seystahl et al. [96]	20 (20)	2016	177Lu-DOTATATE90Y-DOTATOC	1–4 (range) 3 (median)	13.7–27.6 (range) 20.2 (median)	SD 10 PD 10	5.4 (median)	RT 18 ** AE 8 ** Surgery 7 ** CT 6 **	20 (median)	PFS at 6 months in 42%. OS at 12 months in 79%.
Parghane et al. [97]	5 (500)	2019	177Lu-DOTATATE	2–6 (range)	19.86 (mean) 13.28–29.97 (range)	SD 5 §§ PR 3 § PD 2 §	26.25 (mean) 16.65–35.84 (range)	CT 2 ** SSA 2 ** Surgery 1 **	19.4 (mean) 8–36 (range)	Regard to neurological symptomatic response: CR in 2/5, PD in 2/5, and PR in 1/5
Bodei et al. [98]	1 (51)	2011	177Lu-DOTATATE	1–6 * (range)	3.7–29.2 (range) *	SD 1	36 (median) *	SSA 43 * Surgery 35 * Surgery + SSA 30 * CT 11 *	29 (median) * 4–66 (range) *	OS in 68% at 36 months. *
Minutoli et al. [80]	8 (8)	2014	111In-Pentetreotide 90Y-DOTATOC 177Lu-DOTATATE	2–4 (range)	4.8–29 (range)	SD 5 PR 2 PD 1	NA	Surgery 4 NA or naive 2 Surgery + RT 1 Surgery + PRRT 1	4–50 (range)	Significant improvement of clinical condition in 4/8 patients. 111In might be used in cases with a high risk of renal toxicity
Van Essen et al. [99]	5 (22)	2006	177Lu-octreotate	2–4 (range)	14.8–29.6 (range)	PD 3 SD 2	NA	RT + Surgery 3 RT + CT 2	3 (at least)	PRRT could be used if the disease is slowly progressive.
Sabet et al. [100]	1 (1)	2011	177Lu-DOTATE	3	18.7	SD	NA	NA	3	Pain reduction and improved life-quality.
Kreissl et al. [101]	10 (10)	2012	RT + 177Lu-DOTATATE or 177Lu-DOTATOC	1	7.4 ± 0.3	SD 8 PR 1 CR 1	NA	Surgery 9 Surgery + RT 1	13.4 (median) 1.1–17.0 (range)	Increased uptake of 68Ga-DOTA in meningioma after the combined therapy

Legend: FU: follow-up, PFS: progression-free-survival, OS: overall-survival, NA: not-available, SD: stable disease, PD: progression disease, PR: partial response, RT: radiotherapy, CT: chemotherapy, SSA: somatostatin analogue therapy, AE: angiographic embolization, * data from the whole cohort, ** NA if in combination or alone, § molecular imaging evaluation, §§ structural imaging evaluation.

## Supplementary Materials

The following are available online at https://www.mdpi.com/2072-6694/11/10/1412/s1, Figure S1: PRISMA flow-chart for dota-peptide’s diagnostic use, Figure S2: PRISMA flow-chart for dota-peptide’s therapeutic use.
