# Supplementary material for: Theragnostic Use of Radiolabelled Dota-Peptides in Meningioma: From Clinical Demand to Future Applications
Source: Cancers (Basel). 2019 Sep 22;11(10):1412. doi: 10.3390/cancers11101412 (PMC6826849; doi:10.3390/cancers11101412)
Supplement: Supplementary file 1 [file cancers-11-01412-s001.pdf]

Supplementary Materials: Theragnostic Use of Radiolabeled Dotapeptides in Meningioma: From the Clinical Demand to Future Applications.

Riccardo Laudicella, Domenico Albano, Salvatore Annunziata, Diletta Calabrò , Giovanni Argiroffi, Elisabetta Abenavoli, Flavia Linguanti , Domenico Albano, Antonio Vento, Antonio Bruno, Pierpaolo Alongi and Matteo Bauckneht

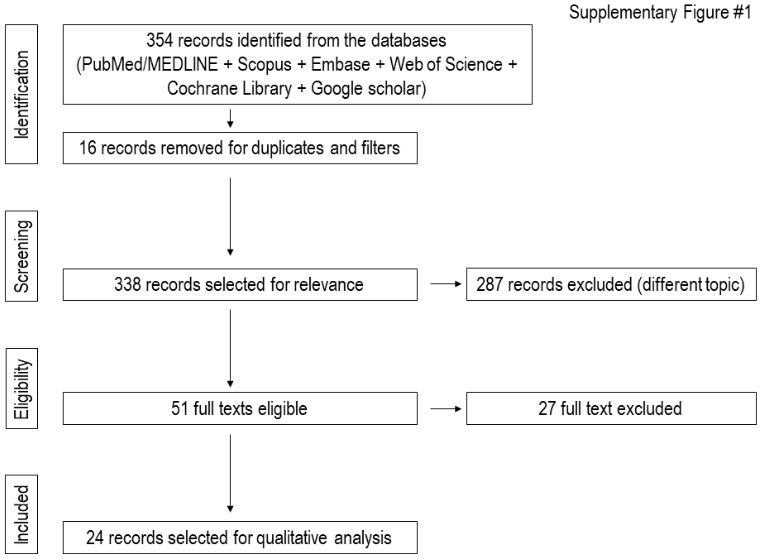

Figure S1. PRISMA flow-chart for dota-peptide's diagnostic use.

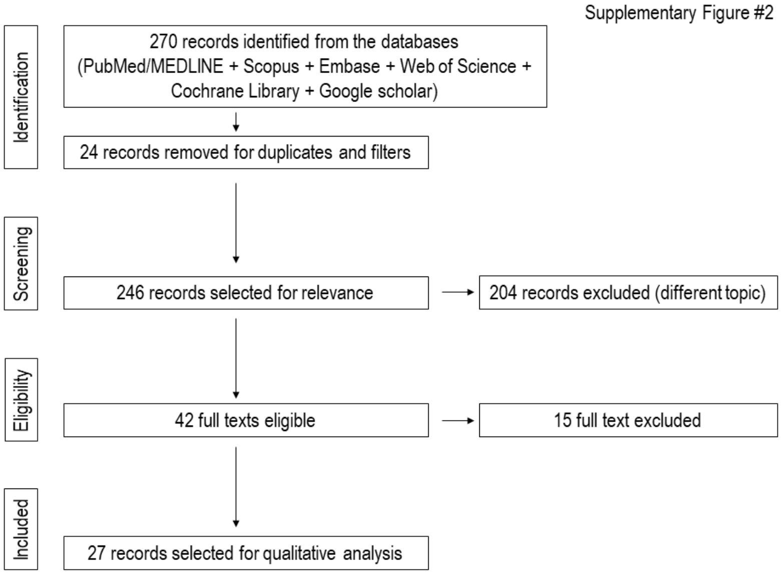

Figure S2. PRISMA flow-chart for dota-peptide's therapeutic use.

© 2019 by the authors. Licensee MDPI, Basel, Switzerland. This article is an open access article distributed under the terms and conditions of the Creative Commons Attribution (CC BY) license (http://creativecommons.org/licenses/by/4.0/).
